# Aorta macrophage inflammatory and epigenetic changes in a murine model of obstructive sleep apnea: Potential role of CD36

**DOI:** 10.1038/srep43648

**Published:** 2017-02-27

**Authors:** Rene Cortese, Alex Gileles-Hillel, Abdelnaby Khalyfa, Isaac Almendros, Mahzad Akbarpour, Ahamed A. Khalyfa, Zhuanghong Qiao, Tzintzuni Garcia, Jorge Andrade, David Gozal

**Affiliations:** 1 Section of Pediatric Sleep Medicine, Department of Pediatrics, Pritzker School of Medicine, Biological Sciences Division, The University of Chicago, Chicago, IL, USA; 2 Center for Research Informatics, The University of Chicago, Chicago, IL, USA.

## Abstract

Obstructive sleep apnea (OSA) affects 8–10% of the population, is characterized by chronic intermittent hypoxia (CIH), and causally associates with cardiovascular morbidities. In CIH-exposed mice, closely mimicking the chronicity of human OSA, increased accumulation and proliferation of pro-inflammatory metabolic M1-like macrophages highly expressing CD36, emerged in aorta. Transcriptomic and MeDIP-seq approaches identified activation of pro-atherogenic pathways involving a complex interplay of histone modifications in functionally-relevant biological pathways, such as inflammation and oxidative stress in aorta macrophages. Discontinuation of CIH did not elicit significant improvements in aorta wall macrophage phenotype. However, CIH-induced aorta changes were absent in CD36 knockout mice, Our results provide mechanistic insights showing that CIH exposures during sleep in absence of concurrent pro-atherogenic settings (i.e., genetic propensity or dietary manipulation) lead to the recruitment of CD36(+)^high^ macrophages to the aortic wall and trigger atherogenesis. Furthermore, long-term CIH-induced changes may not be reversible with usual OSA treatment.

Obstructive sleep apnea is a common and highly prevalent disorder affecting 8–10% of the general population. Obesity constitutes a major risk factor of OSA, the latter being characterized by recurrent upper airway collapse that occurs during sleep, and leads to episodic hypoxemia and sleep fragmentation^1^,^2^,^3^. Both epidemiologic and intervention-based studies have provided conclusive evidence indicating a causative link between OSA and cardiovascular morbidity, independently from associated obesity^4^,^5^,^6^. However, the mechanisms underlying the accelerated atherogenesis that is putatively ascribed to OSA remain elusive. Furthermore, a very recent large-scale multicenter randomized trial suggested that treatment of OSA in patients with moderate to severe disease did not prevent cardiovascular events in those with established cardiovascular disease, suggesting that long-standing OSA may lead to relatively irreversible vascular disease^7^.

Chronic intermittent hypoxia (CIH) during the sleep period, has been used as a useful murine model of OSA, and promotes the presence of increased atherogenesis, particularly in conjunction with other predisposing risk factors such as transgenic ablation of *LdlR* or *ApoE*, or concurrent feeding of a high fat diet^8^,^9^,^10^,^11^,^12^,^13^,^14^. Macrophages are central to the processes mediating atherogenesis, and have been implicated in the initiation and progression of atherosclerosis through intrinsic activation, changes in polarity and phenotype, production of cytokines, and signaling to other cells in the vessel wall^15^,^16^,^17^,^18^,^19^. It is now well established that the presence of a pro-inflammatory M1-like macrophage phenotype is associated with increased plaque burden and adverse outcomes^15^,^16^,^17^,^18^,^19^. Specifically, activation of aortic macrophages through scavenger receptor B3 (CD36) has been shown to mediate atherosclerosis development in other pro-atherogenic disease models^20^,^21^,^22^. Moreover, a novel metabolically active M1-like macrophage phenotype has been recently described in the context of obesity-related adipose tissue inflammation and insulin resistance^23^, whereby positive CD36 surface labeling conclusively identified this metabolic M1 macrophage phenotype. Recent reports have also suggested that epigenetic modifications may determine phenotype and activity profiles of macrophages, and more specifically the pro- or anti- atherogenic profile of macrophages in vascular areas prone to atherosclerosis, suggesting the possibility that epigenetic mechanisms may underlie aspect of macrophage M1 polarization in CIH^24^,^25^,^26^, as well as the potential reversibility of the atherogenic process following treatment^26^.

In the current study, we aimed to expand on our recent findings in which relatively short exposures of up to 6 weeks of CIH promote accumulation of CD36^+^ macrophages in the aorta of exposed wild type mice fed normal chow diet. Here, we extend murine CIH exposures to 20 weeks duration, more closely mimicking the usual chronicity of human OSA clinical disease. Through multi-omic approaches we show that the increased accumulation and proliferation of pro-inflammatory M1-like macrophages in the murine aorta is characterized by a set of differentially expressed protein surface markers, global transcriptional changes and corresponding epigenetic modifications in these cells. Furthermore, transgenic CD36 ablation protects mice from the aforementioned deleterious changes to the vasculature, thus providing mechanistic insights into the potential role of CD36 in CIH-induced atherogenesis. Finally, we examined the effect of treatment, i.e., cessation of CIH, on the reversibility of the morphological and macrophage changes.

## Methods

### Animals

All animal care and experimental procedures were approved by the animal care committee at the University of Chicago (protocol # 72043). All methods were carried out in accordance with The Principles of Laboratory Animal Care (NIH publication no. 85Y23, revised 1996). C57BL/6 J and C57BL/6 J CD36^−/−^ mice (Jackson Laboratory, Bar Harbor, Maine) had unrestricted access to water and regular chow diet, and were housed in standard conditions in a temperature-controlled room (24 ± 2 °C) with 12:12 h light–dark cycles (lights on at 07:00 AM). Transgenic B6.Cg-Tg (ACTB-mRFP1)1F1Hadj/J mice (termed RFP) weighing 22–25 g, were also purchased from Jackson Laboratories (Bar Harbor, Maine). RFP mice express the red fluorescent protein (DsREDT3) under the control of a chicken beta-actin promoter and cytomegalovirus enhancer. Following exposures, mice were sacrificed using CO_2_ intoxication and exsanguination via cardiac puncture. Aortas were perfused with 5–10 ml of ice-cold PBS supplemented with 20IU/ml heparin, dissected and cleaned under the microscope from surrounding adipose and lymphoid tissues.

### CIH exposures

Animals were housed in identical commercially designed chambers (30′′ × 20′′ × 20′′) that can accommodate 24 mice each, and are operated under 12 hour light-dark cycle (Oxycycler model A44XO, Biospherix). A custom oxygen concentration profile for the 12 daylight hours was originally extrapolated from the saturation recordings of an adult patient with moderate to severe obstructive sleep apnea, which consists of 90 sec of 6.1% O_2_ balance nitrogen alternating with 90 sec 21% O_2_ (room air for 12 hours during the light phase. The O_2_ concentration in the chambers was continuously monitored and automatically adjusted throughout the 12 hours of light time (07:00 a.m.–07:00 p.m.). For the remaining 12 hours of nighttime, oxygen concentrations were kept at 21% O_2_. Chronic intermittent hypoxia exposures lasted 20 weeks. Mice in the room air control group were housed in matching cages and exposed to 21% O_2_.

### Flow Cytometry

Detailed protocol is provided as Supplementary Information. Dissected aortas were minced and incubated with an aorta dissociation cocktail. For flow cytometry analysis, cells were fixed and stained with specific antibodies. Data were acquired on a FACS CantoII cytometer using the FACS Diva 5.5 software (BD Biosciences, San Jose, CA) and analyzed by FlowJo software (Tree Star, San Carlos, CA). In all of the experiments, macrophages were identified as CD11b-F4/80 double positive cells.

### Cell isolation

Detailed protocols are provided as Supplementary Information. In brief, macrophages were isolated using magnetic beads with an EasySep positive selection kit (STEMCELL Technologies Inc., Vancouver, Canada). Monocytes were isolated from whole blood using a monocyte enrichment kit (STEMCELL). Bone-marrow derived monocytes were enriched in a similar fashion from cells isolated from perfused femurs and tibias of eight-week old transgenic B6.Cg-Tg (ACTB-mRFP1)1F1Hadj/J mice (termed RFP) male mice and matched wild-type controls.

### Transcriptomics analysis

Total RNA were isolated from aorta macrophages using miRNeasy Mini Kit-column-based system (Qiagen, Valencia, CA, USA). Purified total RNAs were processed for labeling using the Low RNA Input Fluorescent Linear Amplification Kit (Agilent Technologies, Santa Clara, CA) and hybridized with whole-genome mouse Agilent microarrays (8 × 60 K). Microarray data was validated by qRT-PCR in a selected number of loci. Detailed protocols and data analysis methods are provided as Supplementary Information.

### Epigenetic profiling

Detailed protocols are provided as Supplementary Information. Macrophages were isolated from aortas as described above. Immediately after isolation, cells were crosslinked and chromatin immunoprecipitated using the True MicroChip kit (Diagenode, Denville NJ) and antibodies specific for H3K9ac and H3K27me3 histone modifications. Sequencing libraries were prepared using the MicroPlex kit (Diagenode). Libraries were pooled and spike-in PhiX Control v3 (Illumina, San Diego, CA) was added. Clusters were generated and sequenced using a HiSeq2000 instrument (Illumina). Sequencing data was submitted to the GEO database (Accession number: GSE86851). Sequencing data was verified by single locus ChIP-qPCR analysis.

### IHC and Image Processing

Carotid arteries were harvested from several mice in each treatment group and embedded in paraffin. Sections from each carotid artery were designated for H&E staining (at 1,000, 3,000, and 5,000 μm), and other staining protocols, as detailed in Supplementary Information.

### Electric Cell-substrate Impedance Sensing (ECIS)

The ECIS array enables assessment of morphological cell changes, cell locomotion, and other behaviors directed by the cell’s cytoskeleton. As cultured cells attach and spread on the electrode surface, impedance is altered, and serves as a measure for disruption of the endothelial cellular junction. This method is based on measuring non-invasively the frequency-dependent electrical impedance of cell-covered gold-film electrodes along the time course of the experiment. Detailed protocol is provided as Supplementary Information.

### Data Analysis

Results are presented as means ± SD, unless stated otherwise. All numerical data were subjected to statistical analysis using independent Student t tests or analysis of variance followed by post-hoc tests (Tukey) as appropriate. Statistical analyses were performed using SPSS software (version 21.0; SPPS Inc., Chicago, Ill.). For all comparisons, a 2-tailed p < 0.05 was considered to define statistical significance.

## Results

### CIH promotes vascular remodeling, vascular wall inflammation and accumulation and proliferation of bone-marrow derived macrophages in murine aorta

**Figure 1:**
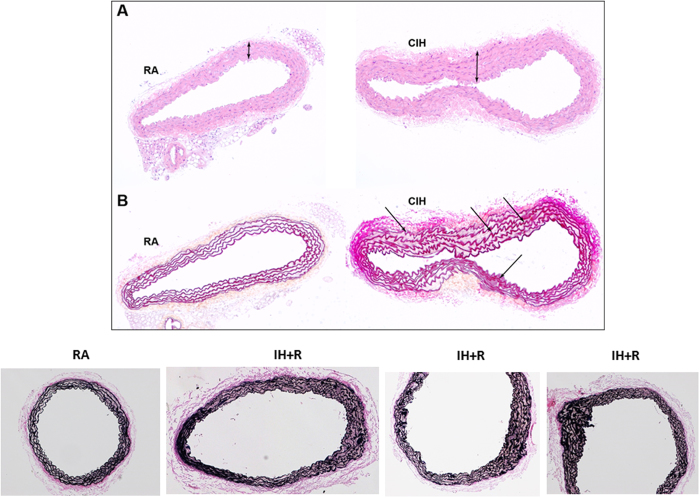
Vascular dysfunction in aortas from mice exposed to CIH. Histological analysis of aortas showing increased IMT (Panel A, top) and disruption of the integrity of elastic laminae (Panel B, bottom). Aortas were collected from mice exposed to 20 week-CIH (CIH, right) and RA controls (RA, left). In addition the bottom panel illustrates the persistent disruption of the elastic laminae in CIH-exposed mice after 6 weeks of normoxic recovery (IH + R) when compared to timed control mice (RA). Images are representative of at least 5 mice/experimental group.

Histological analyses of aortas from mice exposed to CIH demonstrated components of vascular dysfunction, characterized by increased intima-media thickness (IMT) (Fig. 1A) and disruption of the integrity of elastic laminae (Fig. 1B). Consistent with the histological findings, the semi-dry weight of cleaned aortas was significantly higher in CIH mice, a surrogate indicator of hypertrophic changes.

**Figure 2:**
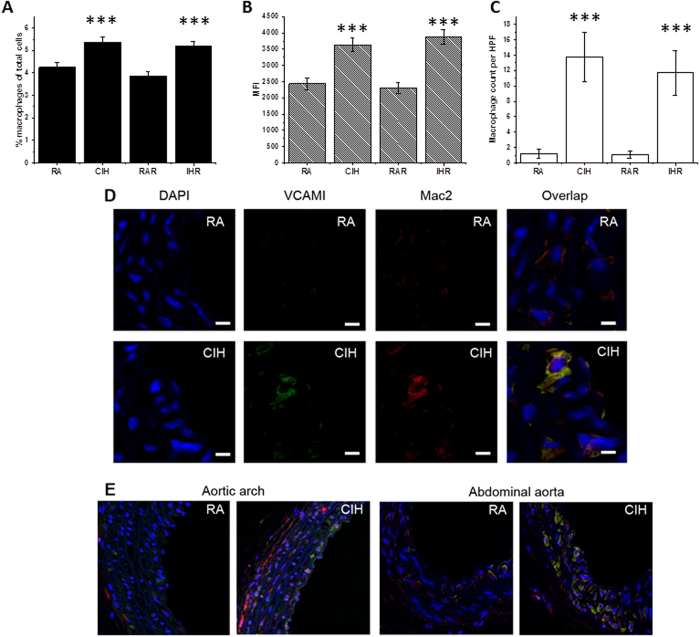
Increased aorta macrophage proliferation in mice exposed to CIH. (**A**) The percentage of macrophages among cells isolated from aorta was significantly higher in CIH-exposed mice than in RA controls, and in CIH-exposed mice after 6 weeks of recovery (IHR) when compared to normoxic time controls (RAR). (**B**) The number of aorta macrophages expressing Ki67 (a marker of macrophage proliferation) was significantly higher in CIH-exposed mice and in IHR mice compared to corresponding controls. (**C**) The count of RFP-derived macrophages was significantly increased in CIH-exposed mice and in IHR mice compared to corresponding controls. (**D**) Confocal microscopy analysis of aorta macrophages in CIH-exposed mice and RA controls. Cells were stained with DAPI (blue fluorescence), and with antibodies against VCAM1 (green fluorescence) and MAC2 (red fluorescence). ***p < 0.04 vs. corresponding control; n = 6/experimental group.

In agreement with a previous study^27^, exposures to CIH in C57BL/6 J mice being fed a regular chow resulted in a 25% increase in the number of aortic macrophages as measured by flow cytometry (4.1% ± 0.1% *vs.* 5.1% ± 0.2%, p = 0.001) (Fig. 2A). In addition, aortic macrophages had increased proliferation rates as indicated by Ki-67 staining (MFI in RA: 2409 ± 260 *vs*. 3783 ± 513 following CIH, p = 0.004; Fig. 2B).

The increased proportion of aortic macrophages under CIH conditions consisted of Ly-6c^high^ (5.9% ± 0.6% *vs.* 8.7% ± 0.9%, p = 0.02), thus indicating that these were myeloid cells originating from the bone-marrow and promoting atheroma formation^28^. Moreover, the putative atheroprotective anti-inflammatory Ly-6c^low^ macrophages^29^ were less abundant in CIH aortas (70.8% ± 1.4% *vs*. 63.2% ± 1.5%, p < 0.001). Similarly, the proportion of anti-inflammatory tissue-resident macrophages^30^, identified here as CD64+ cells, was lower in CIH-exposed aortas (61.7% ± 2.5% vs. 54.6 ± 3%, p < 0.05). To further confirm the notion that bone-marrow recruitment provides a major source of increased macrophage accumulation in aorta wall following CIH exposures, we isolated bone-marrow monocytes from RFP mice and injected into RA- or CIH-exposed WT C57BL/6 J mice. One week after i.v. injection of RFP monocytes, blinded histological analysis of the aortas showed significantly increased homing of RFP-positive cells into CIH aortas (Fig. 2C). Similar to non-injected CIH exposed mice, the locations of RFP monocytes were primarily in the aortic arch and abdominal aorta regions.

To further ascertain aortic macrophage accumulation following CIH, we found similarly increased numbers of macrophages in the aortic wall of C57BL/6 J mice exposed to CIH using confocal microscopy imaging (Fig. 2D). As would be anticipated based on well-established atherosclerosis models^9^,^31^, the most pronounced effects of CIH occurred in vessel regions that are prone to development of atherosclerosis, i.e., aortic arch and lower abdominal aorta (Fig. 2E). Additionally, VCAM-1 expression, an adhesion molecule critical to the atherogenic process^32^, was increased in aortic sections of CIH mice. Interestingly, high magnification images revealed that the increased VCAM-1 expression localized to both macrophages and endothelial cells, reinforcing the concepts on the importance of interactions between activated endothelium and myeloid cells (Supplementary Figure S1). In contrast with nearly complete recovery of macrophage counts when CIH-exposed mice for 6 weeks were returned to normoxic conditions for an additional 6 weeks^26^, return of a subset of mice after 20 weeks of CIH (n = 6) to normoxia for 6 weeks revealed persistent and unaltered increases in aorta macrophage counts, VCAM1, and Ki67 staining (Fig. 2).

### CIH exposed blood monocytes exhibit increased ability to disrupt endothelial barrier *ex-vivo*

**Figure 3:**
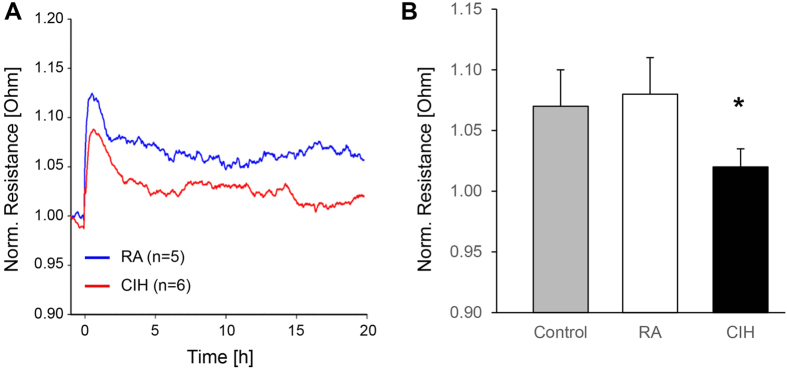
CIH-derived monocytes disrupts the cell culture monolayer. (**A**) ECIS results showing the monolayer electric resistance registered across several time points after exposing the cell monolayer to CIH-derived monocytes (red line) and RA-derived monocytes (blue line). (**B**) Mean electrical resistance at the baseline (only cell monolayer, gray bar), after exposure to RA-derived monocytes (white bar) and after exposure to CIH-derived monocytes (black bar). Electrical resistance is significantly lower after exposure to CIH-derived monocytes comparted to baseline. (p < 0.05). n = 8/experimental group.

Activated monocytes display an increased ability to attach to the vascular lining, penetrate the endothelial barrier and migrate into the intima regions of the vessel. Circulating monocytes from CIH and RA-exposed mice were isolated and added to a monolayer Bend.3 endothelial cell culture *in vitro*, while assessing transcellular layer resistance using ECIS. Of note, the overall percentage of monocytes in peripheral blood and the relative proportions of Ly-6c^high^ monocytes were similar in RA and CIH-exposed mice. However, monocytes from CIH-exposed mice readily disrupted the integrity of the monolayer to a much greater extent than RA-derived monocytes, as evidenced by larger reductions in monolayer resistance, thereby indicating functional activation consistent with the phenotypic changes in aortic macrophages described below (Fig. 3). In support of these findings, significant increases in the expression of genes representing monocyte transmigration processes across vessel wall, such as integrins (*Itgb2*), immunoglobulin superfamily members (*Icam1, F11r, Amica1*), selectins (*Sell*) and chemokine receptors (*Ccr2*)^33^ emerged in the transcriptome of aortic macrophages (see below).

### CIH promotes M1-CD36+ metabolically active macrophage phenotype

Macrophage scavenger receptor B3 (CD36) participates in the uptake of oxLDL and promotes atherogenesis through increased myeloid cell accumulation in the arterial intima^20^. Recently, CD36 has been identified as a novel marker for macrophage activation in the context of metabolic stimuli such as free fatty acids (FFA) or hyperglycemia, rather than by bacterial infection-induced inflammation^23^, indicating a very unique subset of pro-inflammatory macrophages with a more specific role in processes such as atherogenesis. We should remark that CIH is characterized by significant dyslipidemia and metabolic dysfunction as well as by higher circulating levels of FFA^34^. We found that CD36^+^ macrophages were present in significantly higher numbers in CIH aortas (37.6% ± 2.6% in RA *vs.* 48.0% ± 1.7% in CIH, p = 0.007). Next, we tested aortic macrophages for surface expression of a classical M1 marker, CD86^35^, which serves as a co-stimulatory molecule expressed during foam cell formation (along with CD36), and is implicated in the activation of T-cells in atherogenesis^36^. CD86 expression was significantly increased on the surface of aortic macrophages following CIH (17.1% ± 0.9 in RA *vs*. 21.4 ± 1.8 in CIH, p = 0.04). Interestingly, CD11c, a surface marker of M1 macrophages upregulated in adipose tissue macrophages following CIH^37^, was not different between CIH and RA conditions. This finding is consistent with previously published studies regarding lack of CD11c expression on murine aortic macrophages^35^,^38^. To further characterize the sub-populations of macrophages that exhibit increased CD36 surface expression: CD36 expression increased 2.3 fold on CD86^+^ macrophages as compared to CD86^−^, 3.1 fold in Ly-6c^high^ macrophages, and 1.14 fold in CD64^−^ macrophages. Interestingly, proliferation rates, measured by Ki-67, were significantly higher in CD36^+^ *vs.* CD36^−^ cells (2.6 fold increase, p < 0.001). Taken together, these data suggest that CIH induces increased CD36 expression in aortic macrophages, preferentially among proliferating bone-marrow derived M1 cells.

### Aortic macrophages from CIH exposed mice change transcription of genes towards a pro-inflammatory, pro-atherogenic program

**Figure 4:**
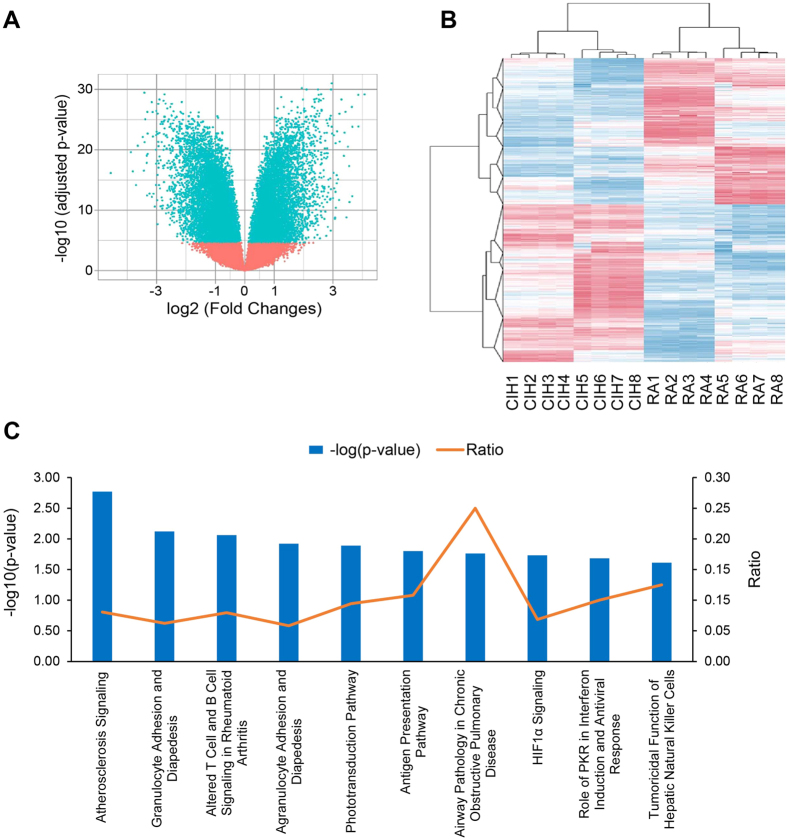
Transcriptomic analysis of CIH-derived aorta macrophages. (**A**) Volcano plot showing differentially expressed transcripts between CIH-derived and RA-derived aorta macrophages (n = 8 per group). We identified 16,343 differentially expressed transcripts between the groups (p < 0.01, blue points). X axis depicts the extent of the difference (log2 Fold Changes) between the groups, whereas Y axis depict the significance of this difference (log10(adjusted p-value)). (**B**) Unsupervised hierarchical clustering of differentially expressed transcripts separate the samples between the two experimental groups. Expression levels are represented as a color gradient in the heatmap, from red (upregulated in CIH) over white (no changes) to blur (upregulated in RA). (**C**) Overrepresented canonical pathways in genes differentially regulated between CIH and RA in aorta macrophages. Blue bars represent the significance of the overrepresentation (−log(p-value)). Orange line depicts the ratio between the number of genes in each canonical pathway and the number of genes differentially expressed in CIH-derived compared to RA-derived aorta macrophages.

Based on the significant changes in a small number of *a priori* known macrophage activation markers, whole genome transcriptomic analyses were performed in CIH and RA-exposed aortic macrophages using microarrays. Bioinformatic analysis revealed significant differences involving ~16,000 genes (Fig. 4A), illustrating the extensive effect of CIH across a multiplicity of gene pathways. Unbiased hierarchical clustering analysis demonstrated a clear segregation of CIH and RA samples (Fig. 4B. These findings were verified using RT-PCR techniques in a subset of genes previously implicated in the pathophysiology of atherosclerosis, such as increased in IL-6, iNOS, and CD36 expression, and reduced ABCA1 expression.

Next, we used a computer algorithm to systematically search PUBMED for studies describing a functional role in atherogenesis for each of the top 20 differentially expressed transcripts in CIH-exposed aortic macrophages. Interestingly, 4 of these 20 genes have been previously identified as atherogenesis-related. Finally, Ingenuity pathway analysis (IPA) was used to study the interactions of the top differentially expressed genes ( ≥ 4-fold difference) to identify their putative biological and functional pathways. This process further indicated that these highly differentially expressed genes were all involved in atherosclerosis and inflammation-related canonical pathways (Fig. 4C). Collectively, these data provide evidence that the differential transcriptional program activated in aortic macrophages *in vivo* following exposures to CIH underlies a pro-atherogenic inflammatory phenotype, even in absence of other predisposing factors such as diet manipulation or genetic propensity.

### CIH promotes epigenetic changes in aortic macrophages corresponding to expression profile

#### Large-scale epigenomic profiling by ChIP-Seq

To explore how CIH exposures affect the epigenetic landscape of the aorta macrophages, we conducted chromatin immunoprecipitation coupled to next-generation sequencing (ChIP-Seq) in macrophages isolated from aortas of mice exposed to CIH or RA conditions for 20 weeks. We investigated two known histone modification marks: acetylation of lysine 9 at histone 3 (H3K9ac) and tri-methylation of lysine 27 at histone 3 (H3K27me3). Presence of such marks have been associated to open and closed chromatin configurations, respectively^39^,^40^.

Given the little amount of cells available per condition (<10,000 cells per mouse), the data generated were limited and presented several challenges for the analysis. Overall, the quality of the sequencing reaction was good with the following yields: CIH-H3K9ac = 5.65 Gbases, RA-H3K9ac = 4.59 Gbases, CIH-H3K27me3 = 2.24 Gbases, and RA-H3K27me3 = 1.55 Gbases (Supplementary Table S4). As detailed in the materials and methods section, we applied several levels of filtration to remove low complexity and blacklisted regions^41^. Additionally, the results from two Chip-seq analysis tools were combined in an attempt to identify the most well-supported differentially bound sites.

For the active mark H3K9ac, we identified 11,685 and 13,854 regions (peaks) showing significant enrichment (adjusted p-value < 0.01) in aorta macrophages isolated from mice exposed to CIH and RA, respectively. Among them, 7,096 and 7,485 were associated to annotated genes. To ensure that the peaks were representative of true epigenetic differences and not artefactual, we further reduced the list of candidate peaks by selecting the 1000 peaks with the highest number of reads for CIH and RA samples, which were associated to 1,486 and 1,482 annotated transcripts, respectively (Supplementary Table S2). Downstream analyses were performed using the reduced list of candidate peaks. In turn, for the inactive mark H3K27me3, we identified 131 and 71 regions (peaks) with significant enrichment (adjusted p-value p < 0.01) in aorta macrophages isolated from mice exposed to CIH and RA, respectively, with 102 and 58 peaks associated to annotated genes. Given the limited number of peaks detected for the repressive mark, no further filters were applied and these candidate lists were used for downstream analysis (Supplementary Table S3).

**Figure 5:**
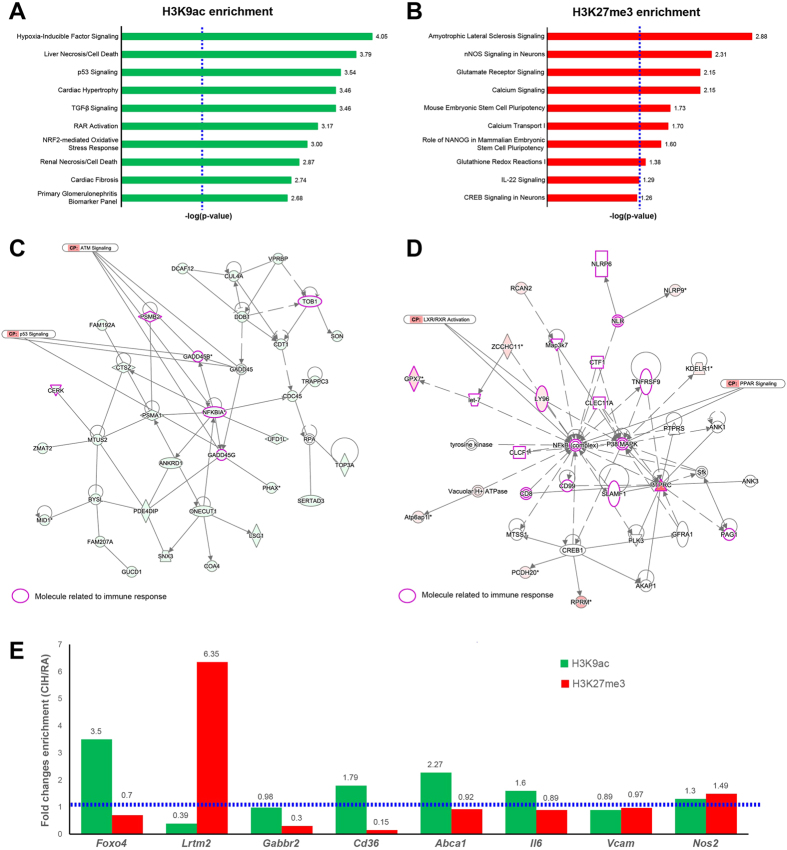
Epigenetic profiling of aorta macrophages from CIH-exposed mice. (**A**) Top ten over-represented canonical pathways among H3K9ac enriched regions. The length of the bar represents the statistical significance from the hypergeometric test expressed as −log(p-value). Vertical dashed blue line represent the significance cutoff (−log(p-value = 0.05) = 1.3). (**B**) Top ten over-represented canonical pathways among H3K27me3 enriched regions. Significance values are expressed as in panel A. (**C**) Example of non-directional gene network of genes bearing H3K9ac marks. Genes bearing the mark are colored green. Molecules related to immune response are highlighted in purple. (**D**) Example of non-directional gene network of genes bearing H3K27me3 marks. Genes bearing the mark are colored red. Molecules related to immune response are highlighted in purple. (**E**) Analysis of candidate genes by single-locus qPCR analysis. Green and red bars represent the CIH/RA fold change enrichment for the H3K9ac and H3K27me3 marks, respectively. Dashed horizontal blue line represents the cutoff value for fold change enrichment (CIH/RA FCE = 1). All data reflect n = 8/experimental condition.

To further investigate functional relations between the genes displaying active or repressive marks, we used a bioinformatics approach (Ingenuity Pathway Analysis) to identify overrepresented biochemical pathways among the genes bearing the epigenetic marks. The software enabled the detection of canonical pathways or lists of molecules that are known to be involved in a particular type of molecular or cellular respond to a given agent (Ingenuity Tox lists), which are significantly over-represented in the candidate lists. We found significant over-representation (p < 0.05, hypergeometric test) of the active mark (H3K9ac) in diverse signaling pathways that are expected to be activated upon hypoxia exposures, such as hypoxia-inducible factor (HIF), p53, and TGF-β signaling (Fig. 5A). Furthermore, we observed significant over-representation of NRF2-mediated oxidative stress response (Fig. 5A). For the repressive mark (H3K27me3), we detected significant over-representation (p < 0.05, hypergeometric test) in pathways related to oxidative stress activation by different pathways to that observed for the active mark (i.e. Glutamate receptor signaling and glutathionine redox reactions), as well as calcium and IL-22 signaling (Fig. 5B). Next, we built unsupervised gene networks depicting the functional interactions between the genes associated to H3K9ac and H3K27me3 peaks. In both cases, we identified networks related to immune responses (Fig. 5C and D). However, whereas networks of genes associated with H3K9ac peaks (active mark) were centered on NF-kB and related to ATM and p53 signaling^42^,^43^,^44^ (Fig. 5C), networks of genes associated with H3K27me3 (repressive mark) were related to anti-inflammatory pathways which are protective against atherosclerosis, such as PPAR/RXR and LXR/RXR activation^45^,^46^ (Fig. 5D).

#### Single locus analysis

We assessed the differences in the two epigenetic marks (H3K9ac and H3K27me3) that occur in aorta macrophages after CIH exposures in 8 candidate genes (Fig. 5E, Supplementary Table S2). We selected 3 genes associated with peaks selected from the ChIP-Seq experiment: *Foxo4* (ChIP-Seq Peak ID: 2102356, increased H3K9ac after CIH; CIH-RA count difference = 113, p-value: 1.26 × 10^−27^), *Lrtm2* (ChIP-Seq Peak ID: 1710745, decreased H3K9ac after CIH; CIH-RA count difference = −125, p-value: 2.51 × 10^−34^), and *Gabbr2* (ChIP-Seq Peak ID: 129982, decreased H3K27me3 after CIH; CIH-RA count difference = −38, p-value:5.36 × 10^−11^). Moreover, we included 5 genes encoding proteins with a known function in macrophage biology and atherosclerosis from which we have previously shown in this study significant increased expression (Bonferroni-adjusted p-value < 0.001) on aorta macrophages after CIH exposures (Supplementary Table S5): *Cd36* (logFC = 1.80), *Abca1* (logFC = 1.12), *Il6* (logFC = 1.38), *Vcam* (logFC = 1.98), and *Nos2* (logFC = 1.07).

We defined CHIP-qPCR-based assays (Supplementary Table S1) for these genes either at regions of reported enrichment for the H3K9ac mark (*Cd36, Abca1, Il6, Vcam and Nos2*) or within the peaks discovered during the ChIP-Seq experiment (*Foxo4, Lrtm2*, and *Gabbr2*). Notably, ChIP-qPCR assays for the *Abca1* and *Il6* genes were also located within the peaks identified by ChIP-Seq: *Abca1* ChIP-Seq peak ID: 1397501, increased H3K9ac after CIH; CIH-RA count difference = 175, p-value = 3.97 × 10^−15^, and Il6 ChI-Seq peak ID: 1505644, increased H3K9ac after CIH; CIH-RA count difference = 106, p-value = 2.26 × 10^−17^.

Enrichment of both epigenetic marks (H3K9ac and H3K27me3) was studied in macrophages isolated from aortas collected from mice exposed to 20 weeks CIH or RA conditions. Figure 5E summarizes the results of the ChIP-qPCR experiments. Enrichment of the epigenetic marks were concordant between the ChIP-PCR and ChIP-Seq results for all the fragments studied: (CIH/RA Fold changes (FC) values were FC_H3K9ac_ = 3.5 and FC_H3K27me3_ = 0.7 for *Foxo4*, FC_H3K9ac_ = 0.39 and FC_H3K27me3_ = 6.25 for *Lrtm2*, FC_H3K9ac_ = 0.98 and FC_H3K27me3_ = 0.3 for *Gabbr2*, FC_H3K9ac_ = 2.27 and FC_H3K27me3_ = 0.92 for *Abca1*, and FC_H3K9ac_ = 1.6 and FC_H3K27me3_ = 0.89 for *IL6*). Furthermore, enrichment of the active epigenetic mark H3K9ac in CIH-exposed mice correlated with increased mRNA expression in the same groups for *Cd36* (FC_H3K9ac_ = 1.79), *Abca1* (FC_H3K9ac_ = 2.27) and *Il6* (FC_H3K9ac_ = 1.6) genes. Our findings support the technical validity of the ChIP-seq results, even when a reduced number of cells was used as starting material. Moreover, our findings suggest a major role for epigenetic regulation throughout histone modifications of the expression of key genes in the pro-inflammatory phenotypic changes in aorta macrophages induced by CIH.

### CD36 mediates the pro-inflammatory changes in aortic macrophages

**Figure 6:**
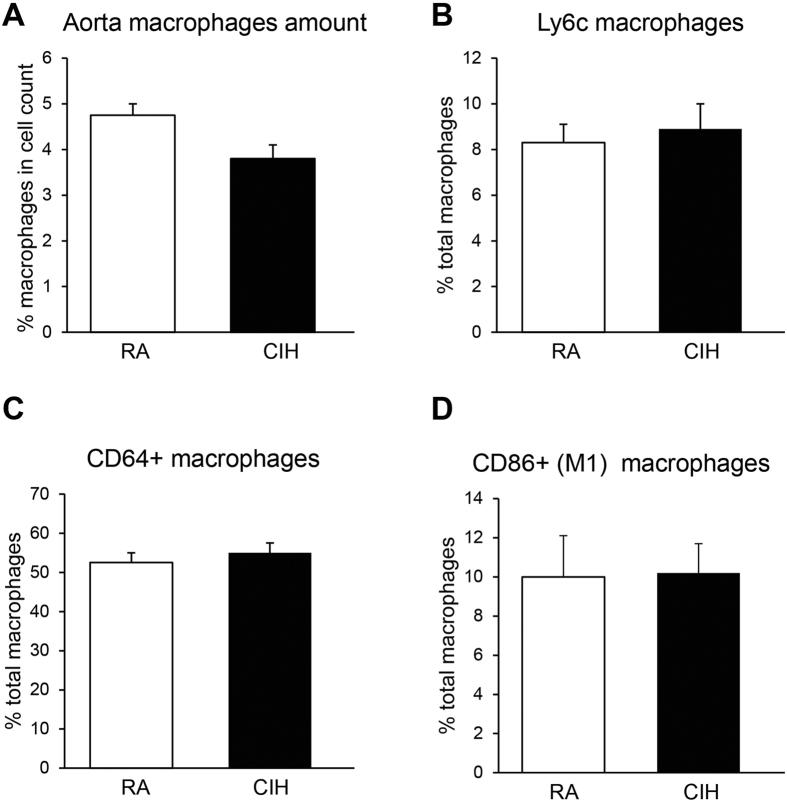
Aorta macrophages in CD36−/− mice. (**A**) In contrast with wild type mice, the percentage of macrophages is lower in CIH-derived than in RA-derived aortas of CD36−/− mice (p < 0.05; n = 6/experimental group). No changes in the proportions of the pro-inflammatory bone marrow-derived (Ly6chigh) (Panel B), tissue-resident (CD64+) (Panel C) and pro-inflammatory M1 (CD86+) (Panel D) cells when compared to RA-exposed CD36−/− mice (p > 0.05; n = 6/experimental group). White and black bars represent RA- and CIH-exposed CD36−/− mice, respectively no changes in the proportions of the pro-inflammatory bone marrow-derived (Ly6c^high^) (**B**), tissue-resident (CD64^+^) (**C**) and pro-inflammatory M1 (CD86^+^) (**D**) cells when compared to RA-exposed CD36^−/−^ mice.

We have previously reported that CIH *in vitro* upregulates transcription of CD36 mRNA in myeloid cells through activation of leukotriene B4 receptor-1 pathways^12^. In the current study, up-regulation of both CD36 mRNA and protein levels in aortic macrophages *in vivo* emerged. To further explore the potential contributory role of CD36 to the aortic macrophage inflammatory process during CIH, we repeated the CIH exposures in mice with a genetic ablation of CD36. CD36^−/−^ mice on a C57BL/6 J background were exposed to CIH and aortas were analyzed for macrophage content and inflammatory phenotype. Remarkably, CD36^−/−^ mice exhibited decreased number of aortic wall macrophages (Fig. 6A), along with no changes in the proportions of the pro-inflammatory bone marrow-derived (Ly6c^high^) (Fig. 6B), tissue-resident (CD64^+^) (Fig. 6C) and pro-inflammatory M1 (CD86^+^) (Fig. 6D) cells when compared to RA-exposed CD36^−/−^ mice. Therefore, these findings suggest a mechanistic link between CD36 expression and CIH induced aortic macrophage inflammation.

## Discussion

This study shows that prolonged chronic exposures to intermittent hypoxia during sleep mimicking OSA elicit recruitment of bone marrow derived macrophages to the aortic wall even when the underlying exposures occur in the absence of a concurrent pro-atherogenic setting, such as genetic propensity or altered dietary intake (e.g. atherogenic or high fat diets). In addition, discontinuation of intermittent hypoxia for a relatively long period is not accompanied by concurrent improvements in aortic wall cellular inflammatory characteristics. Furthermore, the dominant phenotypic polarization of CIH-exposed bone marrow derived macrophages is not only disruptive to the endothelial barrier, but further assumes a unique M1 metabolic profile that is recognizable by the heightened expression of CD36^23^. Transcriptomic approaches further illustrated the extensive effect of CIH on macrophages, whereby multiple previously recognized pro-atherogenic pathways are recruited in a CD36-dependent manner. Finally, assessment of epigenetic changes in aorta macrophages exposed to long-term CIH revealed a complex interplay of histone modifications activating and silencing the expression of genes in functionally relevant biological pathways, such as inflammation and oxidative stress. Noteworthy, we identified repressive and activating marks in functionally related gene networks, but identifying precise pathways leading to the pro-inflammatory phenotypes, revealing that CIH triggers specific epigenetically driven mechanisms during the atherogenic development (Fig. 5).

Before we discuss the potential significance of some of our major findings, some methodological issues deserve comment. First and in line with previous reports^9^, we implemented isolated CIH exposures for 20 weeks without the concomitant imposition of additional risk factors (ApoE or LdlR knockout mice^13^,^22^ or high-fat diet^10^). Although such strategy did not result in development of frank atherosclerotic lesions, we found the presence of significant increases in IMT along with disruption of elastic laminae, both of which are considered as indicative of pro-atherogenic changes^47^,^48^. Indeed, IMT is a widely used non-invasive method in human studies to assess the extent of atherosclerotic burden both in the coronary^49^ and extra-coronary arteries^50^. Increases in IMT have also been described in hypertensive OSA patients^51^ and associated with increased cardiovascular risk. Similarly, elastic laminae are a key component of the normal vascular wall and help transduce pressure waves through the artery wall. Impairment of the normal transduction of the artery pressure wave resulting from disruption of elastic laminae promotes turbulent flow in areas of increased pressure, around bifurcations and curves, and subsequently results in predisposition of these areas to atherosclerosis^52^,^53^. The elastic laminae changes described herein following CIH are compatible with previously reported increases in systemic blood pressure elicited by CIH^54^ and are also present in the context of long-term sleep fragmentation exposures, another hallmark characteristic of OSA^55^. Second, our experiments using monocytes derived from the bone marrow of RFP mice cannot be viewed as conclusively providing evidence that the major source of aorta macrophages originates from the bone marrow in CIH-exposed mice. However, the marked increases in RFP macrophages in the aorta wall after their intravenous administration along with the increased presence of macrophages in the aorta wall bearing markers of their bone marrow origin point to the bone marrow myeloid cells as the major protagonist facilitating CIH-induced aorta inflammatory processes. However, we cannot exclude the possibility that changes in the number of circulating monocytes may have occurred among CIH-exposed mice and that some of the increases in macrophage counts in the aorta wall may be due to such changes. We surmise that such possibility is unlikely, particularly since similar findings were identified in mice exposed to CIH but then allowed to recover in normoxic conditions for 6 weeks. Finally, the changes induced by CIH-exposed circulating monocytes on endothelial barrier electrical impedance do not necessarily indicate that the full process of monocyte activation and transmigration occurred, but nonetheless provide compelling evidence as to the disruption of endothelial barrier integrity, a critical initial event in this process^33^,^56^,^57^. We should also remark that discontinuation of the intermittent hypoxia exposure for a period of 6 weeks did not result in major improvements in either elastic laminae distribution or aorta wall inflammatory cells. Although these findings are preliminary in nature, they clearly raise the possibility that either a 6-week recovery period is insufficient for reversal of 20-week CIH-induced changes, even if such reversal does occur after 6 weeks exposure^26^, or that the prolonged exposures to CIH epigenetically alter inflammatory cells such as macrophages within the aorta to perpetuate pro-atherogenic pathways.

In a previous study, we found that M1 pro-inflammatory macrophage activation in the context of high fat diet or hyperglycemic states involves metabolic pathways that are driven by independent pro-inflammatory and anti-inflammatory pathways, which regulate balance between cytokine production and lipid metabolism^22^. Indeed, we identified that in addition to PPARγ and p62/SQSTM1, two key proteins that limit inflammation in metabolically activated macrophages, cell surface proteins specifically overexpressed by metabolic M1 macrophages included ABCA1, CD36, and PLIN2, i.e., proteins involved in lipid metabolism^22^,^58^. Very interestingly, we detected in the gene network analysis that repressive marks are enriched in PPAR signaling genes (Fig. 5D), suggesting an epigenetic regulation for the PPAR pathway in macrophages upon CIH exposure. The deregulation of lipid metabolism by CIH^59^,^60^,^61^ and the relative similarities between CIH and other metabolic stressors linked to atherosclerosis prompted us to explore the possibility that early CIH may mimic the aforementioned transitions in polarity exhibited by macrophages^27^,^62^. Relatively short-lasting exposures to CIH induced early emergence of an increased population of CD36+ macrophages exhibiting concurrent shift to M1 polarity markers^27^. Based on such findings, we surmise that CIH acts as a major potentiator of the coordinated interplay of multiple pro-inflammatory events that characterize atherosclerosis, including recruitment, activation, and transmigration of bone marrow derived monocytes into M1 macrophages with unique metabolic characteristics^63^,^64^,^65^.

### Epigenetic regulation of pro-inflammatory phenotypes in aorta macrophages

To our knowledge, this is the first time that genome-wide histone modification profiling is conducted in macrophages isolated from murine aortas. This task represented a technological challenge since the amount of recovered cells after the dissection of the mouse aorta is very low (less than 10,000 cells per animal). Despite the technical limitations, we successfully enriched the fractions corresponding to one active (H3K9ac) and one repressive (H3K27me3) chromatin marks in aorta macrophages from mice exposed to CIH and RA paradigms, prepared the libraries and sequenced them by NGS. Single locus analysis of selected candidate genes in a set of independent samples demonstrated that the epigenetic differences were not due to technical artifacts. Noteworthy, the number of peaks obtained for the H3K9ac mark, was considerable higher than those for the H3K27me3. This discrepancy may be explained by the efficiency of the antibody in each IP, but most likely reflects an increased level of active marks and reduced level of repressive chromatin marks in the aorta macrophages, regardless of exposure. In this regard, it has been shown that the H3K27me demethylase Jmjd3 is expressed in migrated macrophages^66^. However, whether the increased number of the active mark H3K9ac in migrated macrophages results from increased acetyltransferase or reduced deacetylase activities remains to be determined. Further experiments are needed to elucidate the molecular mechanisms leading to epigenetic alterations in mouse aorta macrophages, and how they are affected by exposures to CIH conditions.

In contrast with analysis of pre-determined candidate regions, genome-wide analyses enable the discovery of novel regions of epigenetic variations, as well as the identification of pathways and gene networks that are epigenetically modified. Here, we identified an over-representation of active marks (H3K9ac) in CIH-exposed macrophages in genes that are recognized as hypoxia-responsive pathways for transcriptional activation, such as hypoxia-inducible factor, p53, and TGF-β signaling^67^ (Fig. 5). Interestingly, we detected significant over-representation of both the active (H3K9ac) and repressive (H3K27me3) marks in pathways associated to oxidative stress response, though the specific pathways differed. Whereas the H3K9ac was over-represented in NRF2-mediated oxidative stress response, the H3K27me3 was over-represented in glutathione redox pathways (Fig. 5). Nrf2 has been reported as an important transcription factor involved in the induction of the scavenger receptor CD36 in murine macrophages and antioxidant stress genes in atherosclerosis^68^, hence epigenetic-mediated activation of this pathway may be involved in the establishment of the CIH-induced pro-atherogenic macrophage phenotype. In turn, glutathione redox pathway is one of the mechanisms that counteract reactive oxygen species levels^69^, and current findings suggest that it may be epigenetically repressed upon CIH-exposure. Taken together, our results are compatible with the concept that epigenetic regulation of oxidative stress pathways in aorta macrophages of mice exposed to CIH might modulate the response in a pathway specific manner, and may dictate the extent of recovery potential upon discontinuation of CIH. Further molecular and cellular experiments are warranted to unravel the precise regulatory mechanisms and their role in the formation of the atherosclerosis plaque.

## Additional Information

**How to cite this article:** Cortese, R. *et al*. Aorta macrophage inflammatory and epigenetic changes in a murine model of obstructive sleep apnea: Potential role of CD36. *Sci. Rep.* **7**, 43648; doi: 10.1038/srep43648 (2017).

**Publisher's note:** Springer Nature remains neutral with regard to jurisdictional claims in published maps and institutional affiliations.

## Supplementary Material

Supplementary Information

Supplementary Table 2

Supplementary Table 3

Supplementary Table 5
