# Supplementary material for: Aorta macrophage inflammatory and epigenetic changes in a murine model of obstructive sleep apnea: Potential role of CD36
Source: Sci Rep. 2017 Feb 27;7:43648. doi: 10.1038/srep43648 (PMC5327416; doi:10.1038/srep43648)
Supplement: Supplementary Information [file srep43648-s1.pdf]

Aorta macrophage inflammatory and epigenetic changes in a murine model of obstructive sleep apnea: Potential role of CD36.

Rene Cortese*[1,3], Alex Gileles-Hillel*[1], Abdelnaby Khalyfa[1], Isaac Almendros[1], Mahzad Akbarpour[1], Ahamed A. Khalyfa[1], Zhuanghong Qiao[1], Tzintsuni Garcia [2], Jorge Andrade [2], and David Gozal[1]#

(1) Section of Pediatric Sleep Medicine, Department of Pediatrics, Pritzker School of Medicine, Biological Sciences Division, The University of Chicago, Chicago, IL

(2) Center for Research Informatics, The University of Chicago.

(3) Present affiliation: Seven Bridges, Inc. Cambridge, MA

* These authors contributed equally to the work

# Corresponding author:

David Gozal, MD, MBA

Phone: (773) 702-3360; Fax: (773) 926-0756- FAX

Email: dgozal@uchicago.edu

Supplementary Information

Detailed material and methods

Cell isolation

Aortic macrophages: Following digestion and filtering of the aortic cell suspension, as described above, $CD11b^+$ cells were isolated using magnetic beads with an EasySep positive selection kit (STEMCELL Technologies Inc., Vancouver, Canada) per manufacturer's protocol. Isolation purity was ~80%. Average yield per single mouse aorta was 2,000-4,000 cells. Cell pellets were stored at -80°C until processing. For epigenetic profiling, cells were fixed prior to storage as described below.

Monocytes: Whole blood was collected in heparinized tubes and spun for 15 minutes at 1,200 g. After addition of Ficoll (Lymphoprep, STEMCELL Technologies Inc., Vancouver, Canada) leukocytes were separated from the buffy coat fraction. Subsequently, monocytes were isolated with a monocyte enrichment kit (STEMCELL Technologies Inc., Vancouver, Canada) per manufacturer's protocol. Bone-marrow derived monocytes were enriched in a similar fashion from cells isolated from perfused femurs and tibias of each animal.

Bone marrow monocytes: Eight-week old transgenic B6.Cg-Tg (ACTB-mRFP1)1F1Hadj/J mice (termed RFP) male mice were sacrificed and bone marrow cell (BMC) suspensions (1 x 108 cells per mL) were incubated with EasySep™ Biotin Selection Cocktail at 100 µL/mL of cells. Magnetic particles (50 µL/mL) were added and isolated monocytes (400,000 cells/mouse) were counted and injected i.v. in wild type mice exposed to CIH or control conditions.

Flow Cytometry

Dissected aortas were minced and incubated with an aorta dissociation cocktail for 1hr on a shaker at a speed of 35 RPM at 37° C. The cocktail included 450u/ml of Collagenase type I (Worthington Biochemical, Lakewood, NJ, USA), 125 U/ml of Collagenase type XI, 60 U/ml of DNASE I and 60 U/ml of Hyaluronidase (all from Sigma-Aldrich, St. Louis, MO, USA), diluted in PBS + 20mM HEPES. Following incubation, the digestion reaction was stopped with 5% fetal bovine serum in PBS, cells were passed through 100 µm filter, washed and re-suspended for further processing in flow buffer (PBS with 2% FBS). For flow cytometry analysis, cells were fixed with 1% paraformaldehyde solution for 30min at 25°C and washed twice. Non-specific binding was blocked with FcR blocker (Miltenyi Biotec , San Diego, CA) at 1:50 concentration and stained with specific antibodies for analysis. For surface staining we used the following fluoro-conjugated antibodies: CD11b-PB, F4/80-PE/Cy7, CD36-FITC/Alexafluor488, CD11c-APC, Ly-6c-APC / APC-Cy7, CD64-PE, CD86-PerCP/Cy5 (BD Biosciences, San Jose, CA). Isotype controls were employed to establish background fluorescence. Data were acquired on a FACS CantoII cytometer using the FACS Diva 5.5 software (BD Biosciences, San Jose, CA) and analyzed by FlowJo software (Tree Star, San Carlos, CA). In all of the experiments, macrophages were identified as CD11b-F4/80 double positive cells.

Transcriptomics analysis

Total RNA were isolated from aorta macrophages using miRNeasy Mini Kit-column-based system following the manufacturer's instructions (Qiagen, Turnberry Lane, Valencia, CA,

USA). Briefly, purified total RNAs were processed for labeling using the Low RNA Input Fluorescent Linear Amplification Kit (Agilent Technologies, Santa Clara, CA) and hybridized with whole-genome mouse Agilent microarrays (8x60K). Equal quantities of total RNA were labeled with each reaction containing 25 ng of total RNA and 2 µl (34 pg) of RNA spike-in control. The quality of each cRNA sample was evaluated using 2100 Bioanalyzer (Agilent Technologies, Santa Clara, CA). Each sample was hybridized to an Agilent oligonucleotide microarray for all of the 32 independent experiments. The microarray slides were scanned using Agilent dual-laser Microarray Scanner and the digitized images were acquired and processed using Agilent Feature Extraction (FE) software v.12.0.

Background-subtracted intensities were normalized using the quantile method across all microarray experiments. We first corrected microarray expression intensity background using thr norm-exp algorithm with offset value of 50 [1]. The background corrected data were normalized between arrays using cyclic loess method [2]. We applied limma moderated t-test to detect differentially expressed genes, considering the batch effect caused by different chips as covariates in the linear model. P-values were adjusted by Benjamini-Hochberg method [3]. Differentially expressed genes (DEGs) were identified either using false discovery rate (FDR) of 0.05 or log-fold change of 1.5 and FDR of 0.05.

qRT-PCR Validation

qPCR analysis was performed for selected mRNAs using ABI PRISM 7500 System (Applied Biosystems, Foster City, CA). To confirm candidate mRNAs, cDNA was synthesized using 250 ηg of total RNA using a High-Capacity cDNA Archive Kit (Applied Biosystems, Foster City, CA). Ribosomal 18S rRNA, was used as a reference gene to

normalize the expression ratios. All experiments were performed in triplicates. The cycle number (C_t) values were averaged and the difference between the 18S C_t and the gene of interest C_t were calculated to calculate the relative expression of the gene of interest using the $2^{-\Delta\Delta Ct}$ method [4]. The results are presented as fold change.

Epigenomics profiling

Chromatin Immunoprecipitation: Macrophages were isolated from aortas of mice exposed to CIH (n=20) or RA (n=20) conditions for 20 weeks, as described above. Immediately after isolation, cells were crosslinked and chromatin immunoprecipitated using the True MicroChip kit (Diagenode, Denville NJ) and antibodies specific for H3K9ac and H3K27me3 histone modifications, according to manufacturer's instructions. In brief, cells were crosslinked with formaldehyde (1% final concentration) and the reaction was stopped with the addition of glycine to 125 mM final concentration. Cells were lysed and chromatin sheared using the Bioruptor instrument (Diagenode) with the following sonication conditions: 30 seconds ON/30 seconds OFF at high power for 25 minutes. Ten percent of sheared chromatin was reserved as INPUT fraction and the remaining was divided in two aliquots. Each aliquot containing approximately 10,000 cells was immunoprecipitated by incubating with 0.25 µg of antibodies specific to H3K9ac and H3K27ac (Diagenode), respectively. For each IP series, a negative control using an antibody against IgG was ran, as well as a positive control IP reaction using crosslinked and sheared chromatin from approximately 10,000 RAW cells and each corresponding antibody. After 16 hours incubation at 4^{0}C, chromatin was immunoprecipitated using magnetic beads, washed and released by incubating at 65^{0}C for 4 hours. DNA was purified using MicroChip columns

(Diagenode), according to manufacturer's instruction. Performance of the immunoprecipitation reaction was assessed by qPCR at two control loci: *Gapdh* (control for open chromatin) and *Myod1* (control for closed chromatin) using the conditions detailed below. Primer sequences are listed in Supplementary Table S1. Immunoprecipitated DNA was stored at -20^{0}C until further use.

Next-generation sequencing library preparation and sequencing: Sequencing libraries from immunoprecipitated aorta macrophage DNA were prepared using the MicroPlex kit (Diagenode) according to the manufacturer's protocol. In brief, immunoprecipitated DNA was quantified using Picogreen staining protocol (Quant-iT™ PicoGreen® dsDNA Assay Kit, ThermoFisher Scientific, Grand Island, NY) and 10 pg were used as input material. DNA was repaired and end-blunted by enzymatic treatment. Stem-loop adaptors with blocked 5' ends were ligated to the 5' end of the genomic DNA, leaving a nick at the 3' end. The 3' ends of the genomic DNA are extended to complete library synthesis and Illumina-compatible indexes were added through amplification. Libraries were purified using AMPure XP beads protocol (Beckman Coulter, Indianapolis, IN) and quantified using the KAPA Library Quantification Kit for Illumina Sequencing Platforms (Kapa Biosystems, Wilmington, MA). Libraries' fragment size distribution was verified using the Bioanalyzer high sensitivity DNA chip (Agilent Technologies, Santa Clara, CA). Libraries were pooled and spike-in PhiX Control v3 (Illumina, San Diego, CA) was added. Clusters were generated and sequenced using a HiSeq2000 instrument (Illumina).

ChIP-seq data analysis. Sequencing data was submitted to the GEO database (Accession number: GSE86851). Sequencing raw data was managed using the HiSeq Control Software 2.2.38 (Illumina). Primary and secondary analysis was performed using the RTA 1.18.61.0

and CASAVA-1.8.2 software (Illumina), respectively. All data analysis was performed under R environment version 3.1.2 [5]. Raw reads quality control was performed using the *FastQC* software version 0.10.1 Adapter sequences identified by the FastQC tool were trimmed using the *cutadapt* software package version 1.9.1. Trimmed short reads were aligned to the mouse reference genome (mm9 assembly) using the *BWA* short read aligner version 0.7.10. Duplicate reads were removed from the alignments using the *Picard* tools package version 1.121. Previous experiments from the ENCODE project [6] identified genomic regions in areas near centromeres and other areas where a large quantity of reads were consistently aligned in all samples and suggested that they were not included in downstream analyses (blacklisted regions). We excluded such regions from our analyses. To overcome the challenges imposed by the low amount of input chromatin in the ChIP reaction and minimize technical artifacts, we adopted an approach which combined two ChIP-seq analysis tools to corroborate one another and arrive at a consensus set of differentially bound regions. The first tool used is *macs2* version 2.1.0.20140616, a frequently used tool for peak calling. The peaks are reported in several formats, and are assigned a p-value. The reported peaks were filtered based on this p-value ($p<0.01$). The second tool used was *Odin* version 0.0.1, which uses a hidden Markov model to identify differentially bound regions by comparing equivalent windows between two ChIP-seq data alignments. The *bedops* package version 2.4.2 was used to find differentially bound (DB) regions identified by *Odin* which also coincided with putative peaks identified by *macs2*. Many of the remaining DB regions were observed to coincide with repetitive sequences. Therefore, a further filtration was applied based on the 50-mer mappability score track obtained from the UCSC genome browser. All positions in DB regions were required to have a mappability score of 1 which implies that all 50-mer sequences found in the DB

region are uniquely found in the known genome sequence. After filtering, peaks were annotated with the nearest genes using *bedops* package (Supplementary Tables S2 and S3).

Single locus ChIP-qPCR analysis. Immunoprecipitated DNA and input fraction DNA were 1:4 diluted in low TE buffer containing 10 ng/μL salmon sperm DNA and subjected to real-time PCR. The reaction consisted of 1× ABI master mix containing Taq polymerase, dNTPs, SYBR green dye and ROX as passive dye (Life Technologies, Carlsbad, CA, USA) and 200 nM of specific primers (Supplementary Material Table S1). The PCR program started with a *Taq* polymerase activation step (10 min at 95°C) followed by 40 cycles at 95°C for 15 s, 60°C for 1 min and 95°C for 15 s. Data analyses were performed using the 7500 System SDS software version 1.4 (Applied Biosystems). For each sample, the percentage of input recovered in IH and RA samples was calculated as 100*2^[(Ct_{input} – 3,32) – Ct_{sample}]. Fold change enrichment between IH and RA conditions was calculated as FCE=% $Input_{IH}$/% $Input_{RA}$.

Electric Cell-substrate Impedance Sensing (ECIS)

Mouse endothelioma cells (bEnd.3) were grown to confluence on 8W10E electrodes (Applied BioPhysics, Troy, NY) in DMEM supplemented with 2%FBS until signal stabilization. Cells were seeded (30,000 cells) and grown to confluence into ECIS arrays as a single confluent monolayer. ECIS monitors the impedance of small 250-micrometer diameter electrodes used as substrates for cell growth, and as cells grow on the electrode they constrict electrical current flow altering the impedance. The ECIS array enables assessment of morphological cell changes, cell locomotion, and other behaviors directed by the cell's cytoskeleton. ECIS uses 250 μm diameter gold film electrodes deposited on the bottom of cell culture dishes and measures the electrode impedance. As cultured cells

attach and spread on the electrode surface, impedance is altered, and serves as a measure for disruption of the endothelial cellular junction [7–9]. This method is based on measuring non-invasively the frequency-dependent electrical impedance of cell-covered gold-film electrodes along the time course of the experiment. The baseline was established using culture medium (300 µl/well-1) alone and compared with values obtained using electrodes covered with a monolayer of cells in 500 µL medium. Monolayer resistance was recorded at 4,000 Hz for 24 hours after application of 20,000 monocytes per well. A change in the resistance across the cell monolayer indicated increased or decreased tran-cellular permeability. Data were normalized to the average of five recordings prior to application of monocytes and each sample was run in duplicates.

IHC and Image Processing

Carotid arteries were harvested from several mice in each treatment group and embedded in paraffin. Sections from each carotid artery were designated for H&E staining (at 1,000, 3,000, and 5,000 µm), and other staining protocols. The carotid arteries were submitted on numbered cassettes, and their identity was kept on a separate document (blinded). Slides were labeled according to the cassettes to allow for blinding at the time of interpretation and reading. Slides were read using 60× magnification on a Nikon Eclipse TE 2000-U microscope (Nikon Instruments Inc., Melville, NY, USA) and interpreted with calibrated Image-Pro Plus software (version 6.1.0.346, Media Cybernetics Inc., Bethesda, MD, USA). The mean of the measurements done at the 1,000, 3,000, and 5000 µm mark distal to the bifurcation were used in the statistical analyses, with each mouse's CIMT was then averaged to represent the final CIMT measurement from the 2 treatment groups [10].

Supplementary Table S1. ChIP-qPCR primer sequences

Gene	Primer 1 sequence	Primer 2 sequence	Fragment size [bp]
Vcam1	TGCACAGGTAAGAGTGTTCA	GATAGACAGCCCACTAAACG	101
Nos2	TGCCTTCTTAGTTGGATTTC	GAGTGCCTCGTTTGTGTATT	111
Cd36	ACATCCTGAGCATTCCAGT	TGAGGGTGATTTTTCACTTT	108
Abca1	AGATGCTCTCGCTGGTCT	TAGTACAGGGGAGGCATAAA	115
Il6	TTCACAGAGGATACCACTCC	TCTTTTCTCATTTCCACGAT	101
Foxo4	AATAAGAAGTCAGCCACAGG	GAGGTCTGGTCGAGGAAG	108
Lrtm2	CTTAACGATCCCAGGAAAG	CAATCTTCCTGAGTCACCTC	115
Gabbr2	AATGCTTTCACTTGGCTAAC	TCAGACTCCCTCCTCTGTAA	112
Gapdh	GAGGTAGAACTCTCTCGAGA	CCTAGCTTGTGACTAACCTC	103
Myod1	GCTTAGTATCCTCTTCCTGC	CATAGTTCTTTGTCCCCTCC	107

Supplementary Table S2: Selected H3K9ac peaks showing differential enrichment between CIH- and RA-exposed aorta macrophages.

- Provided as separated Excel table

Supplementary Table S3: Selected H3K27me3 peaks showing differential enrichment between CIH- and RA-exposed aorta macrophages.

- Provided as separated Excel table

Supplementary Table S4. Sequencing lane statistics

**Sample**	**Yield (Mbases)**	**# Reads**	**% of raw clusters per lane**	**% of >= Q30 Bases (PF)**	**Mean Quality Score (PF)**
CIH-H3K9ac	5,656	113,111,463	53.77	95.15	37.05
RA-H3K9ac	4,594	91,872,075	43.67	95.12	37.04
CIH-H3K27me3	2,237	44,743,713	32.54	98.37	36.25
RA-H3K27me3	1,554	31,079,018	22.6	98.44	36.26

Supplementary Table S5. Differentially expressed genes (adj $p < 0.01$) in aorta macrophages between 20 week-CIH and RA controls.

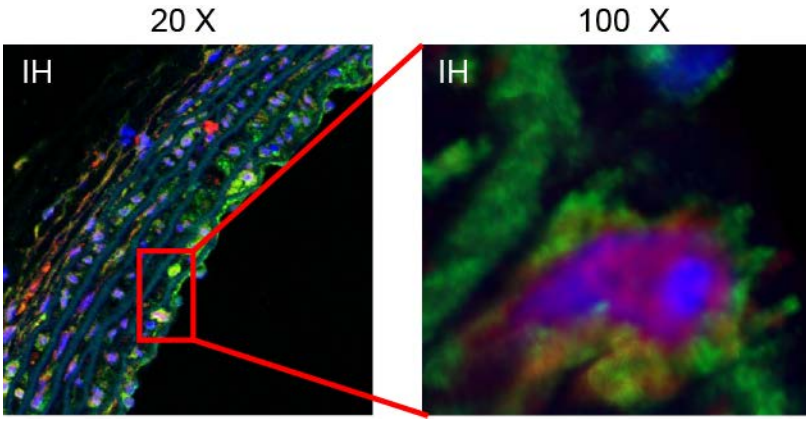

Supplementary figure S1: Interaction between epithelial cells and macrophages in CIH-exposed mice. 20 and 100X magnification of a section of aorta macrophage showing co-localization of macrophages and epithelial cells. Cells were fluorescently labeled and observed in a confocal microscopy as in Figure 2.

References

1. Ritchie, M. E. *et al.* A comparison of background correction methods for two-colour microarrays. *Bioinformatics* **23,** 2700–2707 (2007).
2. Bolstad, B. M., Irizarry, R. A., Astrand, M. & Speed, T. P. A comparison of normalization methods for high density oligonucleotide array data based on variance and bias. *Bioinformatics* **19,** 185–193 (2003).
3. Benjamini, Y. & Hochberg, Y. Controlling the False Discovery Rate: A Practical and Powerful Approach to Multiple Testing. *J. R. Stat. Soc. Series B Stat. Methodol.* **57,** 289–300 (1995).
4. Schmittgen, T. D. & Livak, K. J. Analyzing real-time PCR data by the comparative C(T) method. *Nat. Protoc.* **3,** 1101–1108 (2008).
5. Team, R Core. *R: A language and environment for statistical computing*. (R Foundation for Statistical Computing, 2014).
6. An integrated encyclopedia of DNA elements in the human genome. *Nature* **489,** 57–74 (2012).
7. Keese, C. R., Bhawe, K., Wegener, J. & Giaever, I. Real-time impedance assay to follow the invasive activities of metastatic cells in culture. *Biotechniques* **33,** 842–4, 846, 848–50 (2002).
8. Rahim, S. & Uren, A. A real-time electrical impedance based technique to measure invasion of endothelial cell monolayer by cancer cells. *J. Vis. Exp.* (2011). doi:10.3791/2792
9. Tiruppathi, C., Malik, A. B., Del Vecchio, P. J., Keese, C. R. & Giaever, I. Electrical

method for detection of endothelial cell shape change in real time: assessment of endothelial barrier function. *Proc. Natl. Acad. Sci. U. S. A.* **89,** 7919–7923 (1992).

10. Kupetsky-Rincon, E. A., Li, Q. & Uitto, J. Magnesium reduces carotid intima-media thickness in a mouse model of pseudoxanthoma elasticum: a novel treatment biomarker. *Clin. Transl. Sci.* **5,** 259–264 (2012).
